# Comparative CT Ventricular Morphometrics in Hydrocephalus, Stroke, and Traumatic Brain Injury: A Distortion-Controlled Analysis

**DOI:** 10.3390/jcm15062306

**Published:** 2026-03-18

**Authors:** Andrada-Iasmina Roşu, Laura Andreea Ghenciu, Ovidiu Alin Haţegan, Luminioara Maria Roşu, Emil Robert Stoicescu, Roxana Stoicescu, Emil-Radu Iacob, Sorin Lucian Bolintineanu

**Affiliations:** 1 Doctoral School, “Victor Babes” University of Medicine and Pharmacy Timisoara, Eftimie Murgu Square No. 2, 300041 Timisoara, Romania; iasmina.rosu@umft.ro; 2 Department of Anesthesia and Intensive Care, “Victor Babes” University of Medicine and Pharmacy Timisoara, Eftimie Murgu Square 2, 300041 Timisoara, Romania; 3 Department of Functional Sciences, Discipline of Pathophysiology, “Victor Babes” University of Medicine and Pharmacy Timisoara, 300041 Timisoara, Romania; bolintineanu.laura@umft.ro; 4 Center for Translational Research and Systems Medicine, “Victor Babes” University of Medicine and Pharmacy Timisoara, 300041 Timisoara, Romania; 5 Discipline of Anatomy and Embriology, Medicine Faculty, Vasile Goldis Western University of Arad, Revolution Boulevard 94, 310025 Arad, Romania; 6 Department of Anatomy and Embriology, “Victor Babes” University of Medicine and Pharmacy Timisoara, Eftimie Murgu Square No. 2, 300041 Timisoara, Romania; roxana.iacob@umft.ro (R.S.); s.bolintineanu@umft.ro (S.L.B.); 7 Department of Radiology and Medical Imaging, “Victor Babes” University of Medicine and Pharmacy Timisoara, Eftimie Murgu Square No. 2, 300041 Timisoara, Romania; stoicescu.emil@umft.ro; 8 Research Center for Pharmaco-Toxicological Evaluations, “Victor Babes” University of Medicine and Pharmacy Timisoara, Eftimie Murgu Square No. 2, 300041 Timisoara, Romania; 9 Field of Applied Engineering Sciences, Specialization Statistical Methods and Techniques in Health and Clinical Research, Faculty of Mechanics, ‘Politehnica’ University Timisoara, Mihai Viteazul Boulevard No. 1, 300222 Timisoara, Romania; 10 Department of Pediatric Surgery, “Victor Babes” University of Medicine and Pharmacy Timisoara, 300041 Timisoara, Romania; radueiacob@umft.ro

**Keywords:** hydrocephalus, computed tomography, Evans index, third ventricle width, ventricular morphometrics, stroke, traumatic brain injury, ventriculomegaly

## Abstract

**Background/Objectives:** Ventricular enlargement is a common finding on non-contrast computed tomography (CT) in acute neurological presentations, occurring in hydrocephalus, stroke, and traumatic brain injury. This study evaluated whether routinely available CT-based ventricular morphometric parameters can distinguish hydrocephalus from stroke and traumatic brain injury using initial imaging examinations. **Methods:** This retrospective observational study included 186 adults (68 with hydrocephalus, 64 with stroke, and 54 with TBI) who underwent index non-contrast cranial CT. Quantitative ventricular parameters included Evans index and third ventricle width, alongside temporal horn dilation and periventricular edema. Multivariable logistic regression models were developed to assess diagnostic performance. A primary morphometric model and a distortion-controlled model incorporating midline shift, mass lesions, and hemorrhage burden were analyzed. Discrimination was evaluated using receiver operating characteristic (ROC) curves. **Results:** Patients with hydrocephalus showed significantly greater ventricular enlargement, with higher Evans index and third ventricle width compared with stroke and traumatic brain injury groups. The primary model demonstrated moderate discrimination (AUC 0.71). After adjustment for intracranial distortion variables, model performance improved substantially (AUC 0.91), with balanced sensitivity and specificity at optimized thresholds. Evans index and third ventricle width were the strongest independent predictors. **Conclusions:** CT-derived ventricular morphometrics provide a practical and reproducible approach for differentiating hydrocephalus from stroke and traumatic brain injury on first-presentation CT, supporting objective interpretation in routine neuroimaging practice.

## 1. Introduction

Hydrocephalus represents a pathophysiological condition characterized by abnormal accumulation of cerebrospinal fluid (CSF) within the ventricular system, leading to ventricular enlargement and, in many cases, increased intracranial pressure [1,2,3]. Accurate and rapid identification of hydrocephalus is essential, as delayed diagnosis may result in irreversible neurological impairment, whereas appropriate CSF diversion can be associated with significant clinical improvement [4,5]. In acute and subacute neurological presentations, non-contrast computed tomography (CT) remains the primary imaging modality due to its wide availability, rapid acquisition, and sensitivity for major intracranial pathology [6,7,8].

Despite its central role in emergency neuroimaging, CT assessment of ventricular enlargement is inherently non-specific [9,10,11]. In addition to hydrocephalus, ischemic or hemorrhagic stroke, traumatic brain injury, intracranial mass lesions, and chronic cerebral atrophy can all cause ventricular dilatation [12,13,14]. In these situations, ventricular enlargement may represent secondary phenomena such as mass effect, parenchymal loss, or decreased compliance, rather than an inherent disorder of CSF circulation [15,16]. Moreover, ancillary CT findings commonly associated with hydrocephalus, such as temporal horn dilation, or ventricular asymmetry can also be found in non-hydrocephalic conditions, further reducing the specificity of qualitative picture interpretation [17,18,19,20,21,22].

To improve objectivity in the evaluation of ventricular size, several CT-based morphometric indices have been proposed, such as the Evans index, third-ventricle width, frontal–occipital horn ratio, and measurements of temporal horn dilatation [23,24]. These parameters stand out because they are easily extracted from standard axial CT images and do not require specialized software or complex post-processing [25,26,27]. While such measures are frequently applied in clinical practice, their diagnostic performance has largely been studied in isolation or within selected hydrocephalus cohorts, often without systematic comparison to common radiological diagnostics encountered in routine emergency imaging [28,29,30].

As a result, the extent to which CT-derived ventricular morphometrics can reliably distinguish hydrocephalus from other causes of ventricular enlargement remains incompletely defined. This limitation is clinically relevant, as radiologists are often required to interpret a single initial CT examination, frequently without access to prior imaging or definitive clinical information [31,32,33].

The objective of the present study was to investigate whether a limited set of CT-based ventricular morphometric features can discriminate hydrocephalus from two major neurological diagnostics, stroke and trauma, using first-presentation non-contrast CT examinations. We aimed to characterize baseline CT findings across these diagnostic groups, quantify differences in key ventricular measurements, and evaluate the diagnostic performance of these parameters using multivariable predictive modeling. By focusing exclusively on routinely acquired CT data, this study seeks to define a pragmatic and reproducible morphometric framework applicable to everyday neuroimaging practice.

## 2. Materials and Methods

### 2.1. Study Design

This retrospective observational study evaluated consecutive adult patients who underwent non-contrast cranial CT for acute neurological symptoms and were subsequently categorized into one of three diagnostic groups: hydrocephalus, stroke, or traumatic brain injury. The period for collection was between January 2023 and December 2024. The study was designed to assess whether routinely available CT-based ventricular and parenchymal measurements could distinguish hydrocephalus from common radiological diagnostics encountered in emergency and inpatient neuroimaging practice.

Only the first CT examination obtained during the index clinical encounter was included for each patient, in order to reflect the initial diagnostic scenario and to avoid bias introduced by therapeutic interventions or disease evolution. Patients with prior neurosurgical intervention visible on the index CT were included only if the examination represented the first available CT for that encounter. Cerebrospinal fluid diversion devices were recorded as imaging findings but were not used to define hydrocephalus status.

Hydrocephalus cases corresponded to patients with a documented clinical diagnosis of hydrocephalus established during routine multidisciplinary care, supported by clinical presentation and neuroimaging interpretation consistent with disturbance of cerebrospinal fluid circulation. Stroke and trauma groups were selected as comparator groups to represent frequent causes of secondary ventricular enlargement and intracranial distortion encountered in acute neuroimaging settings.

### 2.2. Imaging Acquisition

All CT examinations were performed using multi-detector CT scanners (GE Healthcare, Chicago, IL, USA) with standard institutional protocols for non-contrast head imaging. Axial images were reconstructed with slice thicknesses ranging from 3 to 5 mm, with multiplanar reconstructions available where necessary to facilitate ventricular measurements. No contrast-enhanced images were used for morphometric analysis, ensuring measurement uniformity across all diagnostic groups.

### 2.3. Imaging Assessment and Variables

CT images were reviewed for ventricular size, configuration, and associated intracranial findings. Measurements and categorical assessments were derived exclusively from the index CT and recorded using predefined criteria.

Ventricular morphometric variables included the Evans index, calculated as the ratio between the maximum width of the frontal horns of the lateral ventricles and the maximal internal diameter of the skull at the same level, and the maximum transverse width of the third ventricle measured in millimeters. Temporal horn dilation was coded as a binary variable based on visible enlargement of the temporal horns beyond their expected anatomical configuration on axial CT images. Both unilateral and bilateral dilation were considered positive findings. No fixed linear diameter threshold was applied, reflecting routine radiological interpretive practice. Periventricular edema was defined as periventricular hypoattenuation adjacent to the lateral ventricle signal consistent with transependymal cerebrospinal fluid flow.

Associated intracranial findings included presence of midline shift, mass lesions, intracerebral hemorrhage, subdural hematoma, epidural hematoma, subarachnoid hemorrhage, intraventricular hemorrhage, contusions, and skull fractures. Markers of chronic brain injury, such as cerebral atrophy and small-vessel disease (leukoaraiosis or microangiopathy), were recorded when explicitly described on CT. Cerebral atrophy was coded as a binary variable (present or absent) based on generalized sulcal widening and ventricular prominence compatible with chronic parenchymal volume loss. Descriptive severity grading was documented but not incorporated into regression modeling. Individual hemorrhagic findings (intraparenchymal, intraventricular, subarachnoid, subdural, and epidural hemorrhage) were aggregated into a composite hemorrhage burden variable to represent overall intracranial bleeding severity within adjusted predictive models. All binary variables were strictly coded as present or absent. No imputation was performed for missing data.

Ventricular morphometric measurements were independently performed by two observers blinded to diagnostic classification. Inter-observer agreement was assessed using intraclass correlation coefficients for continuous variables and Cohen’s kappa for categorical assessments.

### 2.4. Outcome Definition

The primary outcome was diagnostic group membership (hydrocephalus versus non-hydrocephalus). Diagnostic group assignment was determined using a composite clinical reference standard based on review of the electronic medical record, radiology reports, and the final discharge diagnosis recorded during the index hospitalization. Hydrocephalus cases corresponded to patients with a documented clinical diagnosis of hydrocephalus established by the treating neurology or neurosurgery team during routine clinical care. This diagnosis was based on the overall clinical presentation together with neuroimaging interpretation consistent with disturbance of cerebrospinal fluid circulation and, when clinically indicated, neurosurgical management decisions such as cerebrospinal fluid diversion procedures, including external ventricular drainage or ventriculo-peritoneal shunting. Stroke cases corresponded to patients with a clinical diagnosis of stroke established by the treating neurology service on the basis of compatible neurological deficits together with CT findings consistent with cerebrovascular injury. Traumatic brain injury cases corresponded to patients with documented head trauma and CT findings consistent with traumatic intracranial injury, such as cerebral contusions, traumatic intracranial hemorrhage, or skull fractures, as recorded in the radiology report and clinical documentation. Ventricular enlargement in routine radiological interpretation was assessed qualitatively and was not defined using a specific Evans index threshold. Evans index thresholds and other morphometric measurements were not used as diagnostic criteria for group classification; instead, all morphometric variables were measured post hoc from the index CT using standardized definitions.

### 2.5. Statistical Analysis

Continuous variables are summarized as means ± standard deviations, and categorical variables as frequencies and percentages. Group comparisons were conducted using analysis of variance (ANOVA) for continuous variables and chi-square tests for categorical variables. Statistical significance was defined as a two-sided *p* value < 0.05.

For classification analyses, stroke and trauma cohorts were pooled into a single comparator group. The primary modeling framework was designed as a binary classification problem (hydrocephalus vs. non-hydrocephalus) to approximate this real-world decision context. Candidate predictors were selected a priori based on radiological and clinical relevance. Ventricular morphometric parameters included Evans index and third ventricle width (continuous variables), as well as temporal horn dilation and periventricular edema (binary variables). Contextual structural modifiers included patient age and cerebral atrophy.

Three prespecified logistic regression models were constructed. The primary model incorporated ventricular morphometrics alongside contextual variables. A reduced morphometrics-only model was developed to isolate the discriminatory contribution of ventricular expansion parameters. A distortion-controlled model extended the primary model by incorporating imaging markers of intracranial distortion and mass effect, including midline shift, mass lesion presence, and intracranial hemorrhage burden. Sensitivity analyses were additionally performed by repeating the classification models separately for hydrocephalus versus stroke and hydrocephalus versus traumatic brain injury. Additionally, an exploratory sensitivity analysis excluding patients with cerebrospinal fluid diversion devices was performed.

Because the Evans index has a narrow physiological range, it was scaled and reported per 0.1-unit increase to improve interpretability. Given evidence of near-separation driven primarily by ventricular morphometric variables, penalized (ridge) logistic regression was employed to stabilize coefficient estimates and mitigate effect size inflation. Effect measures are expressed as odds ratios with bootstrapped 95% confidence intervals.

Model discrimination was assessed using receiver operating characteristic (ROC) curve analysis, with area under the curve (AUC) reported for each model. Calibration was evaluated using the Brier score. Classification accuracy, sensitivity, and specificity were calculated at both the conventional 0.50 probability threshold and the Youden index-optimized threshold to reflect clinically relevant decision trade-offs. Differences in discrimination between predictive models were formally evaluated using the DeLong test for correlated ROC curves. All statistical analyses were performed using Python (version 3.11) and SPSS software (version 29; IBM Corp., Armonk, NY, USA).

## 3. Results

### 3.1. Study Population

The final study cohort consisted of 186 patients: 68 with hydrocephalus, 64 with stroke, and 54 with traumatic brain injury. Baseline demographic characteristics are summarized in Table 1.

Patients from the trauma group were, on average, younger (59.2 ± 19.7 years) than those with stroke (66.9 ± 11.1 years) and hydrocephalus (61.7 ± 7.6 years). However, the group showed a broader age distribution.

CSF diversion devices were present in 47.1% of hydrocephalus patients, compared with fewer than 7% of stroke and trauma patients. Within the hydrocephalus group, both external ventricular drainage (20.6%) and ventriculo-peritoneal shunting (26.5%) were commonly used.

### 3.2. Baseline CT Characteristics and Associated Imaging Findings

Baseline CT findings demonstrated substantial overlap between diagnostic groups, particularly with respect to secondary imaging features related to hemorrhage, mass effect, and chronic brain injury (Table 2). Ventricular and periventricular features commonly associated with hydrocephalus were not exclusive to this group and were also observed in stroke and trauma patients, albeit with differing frequencies.

Periventricular edema was most frequent in the hydrocephalus cohort, affecting 44 patients (64.7%), but was also present in a considerable proportion of stroke (40.6%) and trauma cases (48.1%). Hemorrhagic findings showed marked group-specific distributions. Intracerebral hemorrhage was rare in hydrocephalus (1.5%), more frequent in stroke (9.4%), and common in trauma (42.6%). Subdural hematomas were predominantly a traumatic finding, occurring in 35 trauma patients (64.8%), compared with only 1 hydrocephalus patient (1.5%) and 3 stroke patients (4.7%). Epidural hematomas were almost exclusively traumatic (27.8%), with only a single case observed in the stroke group and none in hydrocephalus. Subarachnoid hemorrhage followed a similar pattern, being present in 33.3% of trauma patients, absent in the stroke cohort, and rare in hydrocephalus (1.5%). Intraventricular hemorrhage was also uncommon in hydrocephalus, while occurring in 6.3% of stroke and 18.5% of trauma patients.

Cerebral atrophy was present in 39.7% of cases in the hydrocephalus group and 43.8% in the stroke group, while less commonly present in trauma (7.4%). When present, cerebral atrophy in all groups was most often mild to moderate in severity.

Markers of chronic small-vessel disease were common especially across non-hydrocephalus groups, being present in 48.4% of stroke and 59.3% of trauma patients, compared with 32.4% in hydrocephalus. Midline shift was predominantly a feature of trauma (44.4%) and was uncommon in hydrocephalus (5.9%).

Statistically significant between-group differences were observed for age, sex distribution, and hemorrhagic imaging features (all *p* < 0.05). In contrast, CSF diversion prevalence did not demonstrate a significant association with diagnostic group membership.

Atrophy severity percentages were calculated among patients with atrophy.

### 3.3. Primary CT Morphometric Analysis

**Figure 1:**
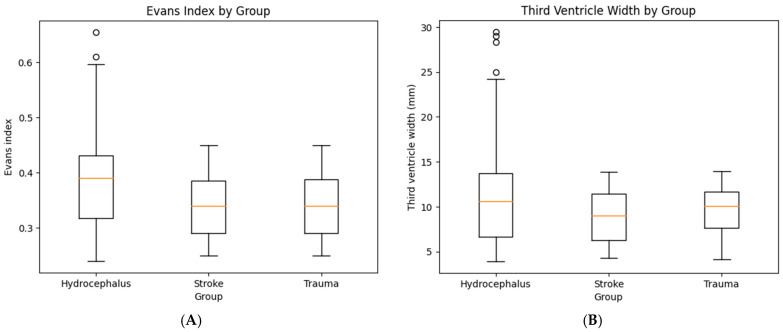
CT-based ventricular morphometrics by group. (**A**) Evans index by group. Box-and-whisker plot showing the distribution of the Evans index across the three study groups. The hydrocephalus group demonstrates a markedly higher median Evans index and wider interquartile range compared with stroke and trauma groups. (**B**) Third ventricle width by group. Box-and-whisker plot illustrating third ventricle width (mm) across the same groups. Patients with hydrocephalus show substantially increased third ventricle diameters relative to stroke and trauma groups.

The primary analysis focused on CT-derived ventricular morphometric parameters hypothesized to distinguish hydrocephalus from stroke and trauma. Patients with hydrocephalus demonstrated marked ventricular enlargement, with a mean Evans index of 0.43 ± 0.07, exceeding the conventional threshold for pathological ventricular dilation. In contrast, Evans index values in the stroke and trauma groups remained substantially lower. Similarly, third ventricle width was significantly greater in the hydrocephalus group, with a mean value of 22.6 ± 5.8 mm, compared with markedly smaller dimensions in both the stroke and trauma groups. Temporal horn dilation and periventricular edema were frequent, but non-specific. Temporal horn dilation also demonstrated a substantial overlap across diagnostic groups and was frequently unilateral in stroke and trauma cases. The distribution of CT morphometric parameters across diagnostic groups is summarized in Table 3. Ventricular morphometric differences across diagnostic groups are illustrated in Figure 1. Patients with hydrocephalus demonstrated markedly higher Evans index values and greater third ventricle widths compared with both stroke and trauma groups.

Inter-observer agreement was excellent for linear ventricular measurements, with ICC values of 0.91 for Evans index and 0.88 for third ventricle width.

### 3.4. Diagnostic Performance of CT Morphometric Parameters

The primary model (Evans index, third ventricle width, temporal horn dilation, periventricular edema, age, and atrophy) demonstrated moderate discrimination (ROC AUC 0.71) and acceptable calibration. At a probability threshold of 0.50, overall accuracy was 0.68, with high specificity but lower sensitivity. When applying the Youden-optimized threshold, sensitivity increased to 0.69 with a specificity of 0.76. Performance metrics for the primary model are presented in Table 4.

The reduced model included ventricular morphometrics only (Evans index, third ventricle width, temporal horn dilation, periventricular edema) and showed similar discrimination (ROC AUC 0.70). As expected, removing age and atrophy minimally altered performance.

To address whether ventricular changes could be explained by secondary deformation due to mass effect or hemorrhage, a distortion-controlled model added midline shift, mass lesion presence, and a composite hemorrhage burden indicator. This model demonstrated substantially improved discrimination (ROC AUC 0.91) and better calibration (Brier 0.11). At the default 0.50 threshold, accuracy was 0.83 with a sensitivity of 0.69 and specificity of 0.91; at the Youden threshold (0.38), sensitivity and specificity were balanced (0.84/0.84).

Within the multivariable classification framework (Table 5), ventricular morphometric parameters demonstrated the strongest independent association with hydrocephalus. The Evans index emerged as the dominant predictor: when scaled per 0.1-unit increase, higher values were associated with a significant increase in the odds of hydrocephalus (OR 1.40). Third ventricle width also retained independent diagnostic value, with each millimeter increase associated with higher odds of hydrocephalus (OR 1.08). Among ancillary ventricular features, periventricular edema demonstrated a positive association with hydrocephalus (OR 1.85). Temporal horn dilation did not independently predict hydrocephalus in the multivariable model (OR 0.78). This could reflect substantial overlap across diagnostic groups.

Contextual variables demonstrated secondary contributions, with age showing a modest inverse association with hydrocephalus (OR 0.98 per year). Cerebral atrophy itself did not retain independent predictive value after adjustment (OR 0.96), indicating collinearity with age and ventricular size metrics rather than direct diagnostic specificity. After adjustment for ventricular morphometrics, atrophy primarily acted as a contextual modifier rather than an independent discriminator.

In the distortion-controlled model, imaging markers of intracranial deformation were strongly associated with non-hydrocephalus diagnoses. Midline shift (OR 0.15) and hemorrhage burden (OR 0.08) markedly reduced the likelihood of hydrocephalus, while mass lesions showed a weaker, non-significant association.

Formal comparison of ROC curves using the DeLong test demonstrated that the distortion-controlled model showed significantly higher discrimination than the primary morphometric model (ΔAUC = 0.20, *p* < 0.001).

Sensitivity analyses were performed by repeating the classification models separately for hydrocephalus versus stroke and hydrocephalus versus traumatic brain injury. The distortion-controlled model maintained strong discriminative performance in both comparisons, with an AUC of 0.88 for hydrocephalus versus stroke and 0.93 for hydrocephalus versus traumatic brain injury. Additionally, an exploratory sensitivity analysis excluding patients with cerebrospinal fluid diversion devices yielded similar results, with the distortion-controlled model demonstrating comparable discrimination (AUC 0.88) to the primary analysis (AUC 0.91).

## 4. Discussion

In this study, we demonstrate that a limited set of routinely available CT-derived ventricular morphometric parameters can distinguish hydrocephalus from stroke and traumatic brain injury on first-presentation non-contrast CT. The predictive modeling approach was employed to formally quantify the discriminative value of CT-based ventricular morphometrics in differentiating hydrocephalus, rather than to propose a diagnostic classifier for standalone clinical use.

Ventricular enlargement following traumatic brain injury or cerebrovascular insult may arise from a variety of mechanisms, such as true CSF circulation disturbance, parenchymal volume loss, mass effect, or secondary obstruction of CSF pathways [34,35]. This diagnostic ambiguity has been particularly highlighted in the context of post-traumatic ventriculomegaly, where the differentiation between compensatory ventricular enlargement and active hydrocephalus remains unresolved in many cases [36]. In their comprehensive review, De Bonis and colleagues emphasize that ventricular widening after trauma does not necessarily reflect a hydrocephalic process and that heterogeneity in diagnostic criteria contributes to the widely variable reported incidence of post-traumatic hydrocephalus, which ranges from less than 1% to more than 50% in different series [37]. Patients with post-traumatic ventriculomegaly rarely present with the classic Hakim–Adams triad and instead typically exhibit nonspecific symptoms related to the primary brain injury, such as impaired consciousness or arrested neurological recovery. This clinical overlap limits diagnostic specificity and reinforces the need for objective imaging-based markers [38]. This uncertainty is further compounded in routine CT imaging, where overlapping secondary features, such as hemorrhage, mass effect, and age-related atrophy, can mimic or mask true CSF circulation disturbance. Consistent with this conceptual framework, our study demonstrated substantial overlap in non-morphometric CT findings across hydrocephalus, stroke, and trauma groups, while quantitative ventricular morphometrics provided more reliable separation. The rationale for using simple, quantitative ventricular markers is further supported by prior work in adjacent diagnostic contexts [39]. In a large MRI-based study, Cagnin et al. demonstrated that a simplified callosal angle measure could accurately differentiate idiopathic normal-pressure hydrocephalus from neurodegenerative dementias, achieving excellent diagnostic performance and high inter- and intra-rater reliability [40].

These results reinforce the notion that hydrocephalus is characterized by a global pattern of ventricular expansion, in contrast to stroke- or trauma-related ventricular changes, which are often regional, asymmetric, or secondary to parenchymal injury. Importantly, temporal horn dilation and periventricular edema, often regarded as supportive signs of hydrocephalus, were also observed in a substantial proportion of non-hydrocephalus cases, showing the limited specificity of individual qualitative features when interpreted in isolation.

The importance of distinguishing hydrocephalus from secondary ventriculomegaly was highlighted by Marmarou et al., who showed that although ventriculomegaly was common after severe head injury, only a subset of patients demonstrated true hydrocephalus when CSF dynamics were assessed [41]. They proposed combining ventricular size metrics with CSF physiological parameters to separate pressure-related hydrocephalus from atrophic enlargement, with implications for shunt selection. This distinction was later reinforced by Guyot and Michael, who reported superior shunt responsiveness in symptomatic hydrocephalus compared with atrophic ventriculomegaly [42]. In our context, these data underscore the value of objective CT morphometrics as an early imaging surrogate when CSF testing is unavailable.

The primary model demonstrated moderate discriminative performance. Classification accuracy was driven primarily by high specificity, while sensitivity remained lower at the default probability threshold, indicating greater rule-in than screening capability. Sensitivity improved following threshold optimization, supporting the role of ventricular morphometrics as an adjunctive interpretive tool rather than a standalone screening test.

### 4.1. Clinical Implications

The clinical relevance of this work lies in its focus on first-presentation non-contrast CT, which is often the only imaging available during initial evaluation. In this setting, radiologists must frequently decide whether ventricular enlargement is most probable attributable to hydrocephalus or represents a secondary phenomenon related to stroke, trauma, or atrophy. The proposed morphometric framework provides objective, reproducible measurements that can complement qualitative assessment and potentially reduce diagnostic uncertainty.

Importantly, the aim of this study was not to replace clinical judgment or advanced imaging, but rather to provide a pragmatic quantitative approach that supports early differentiation and appropriate triage. In patients with equivocal CT findings, such information may help guide further imaging, neurosurgical consultation, or closer clinical monitoring.

### 4.2. Limitations

Several limitations should be acknowledged. First, the retrospective design introduces potential selection bias and limits control over imaging acquisition parameters and clinical characterization. Second, morphometric assessment was restricted to first-presentation CT examinations, precluding evaluation of temporal ventricular evolution or delayed post-injury hydrocephalus. Ventricular morphology in stroke and traumatic brain injury may be confounded by mass effect, hemorrhage, or midline shift, potentially influencing linear measurements despite distortion-adjusted modeling. Third, measurements were derived from CT rather than MRI, which may limit anatomical precision and sensitivity to subtle parenchymal or CSF flow alterations. The analysis focused on a pragmatic set of linear ventricular indices and did not incorporate advanced morphometric markers such as callosal angle, volumetric segmentation, or DESH-pattern assessment. Temporal horn dilation was assessed as a binary qualitative variable without laterality stratification or linear thresholding, which may have contributed to overlap across diagnostic groups. Finally, imaging findings were not correlated with cerebrospinal fluid dynamic testing or post-shunt response, limiting physiological validation.

### 4.3. Future Directions

Future work should focus on external validation of the proposed model, assessment of inter-rater reliability in broader clinical settings, and exploration of automated or semi-automated measurement techniques. Integration of longitudinal imaging and clinical outcomes may further refine the distinction between active hydrocephalus and chronic ventricular enlargement.

## 5. Conclusions

CT-based ventricular morphometric analysis provides a practical and reproducible approach to distinguishing hydrocephalus from stroke- and trauma-related ventricular enlargement on first-presentation CT. Despite substantial overlap in secondary imaging findings, quantitative ventricular metrics demonstrate meaningful discriminative value. These findings support the role of structured CT morphometrics as an adjunct to qualitative interpretation in routine neuroimaging practice.

## Figures and Tables

**Table 1:** Baseline demographics of the study groups.

Characteristic	Hydrocephalus (*n* = 68)	Stroke (*n* = 64)	Trauma (*n* = 54)	*p*-Value
Age, years (mean ± SD)	61.7 ± 7.6	66.9 ± 11.1	59.2 ± 19.7	0.005
Male sex, *n* (%)	32 (47.1)	37 (57.8)	38 (70.4)	0.03
Race/ethnicity				
White/European, *n* (%)	67 (98.5)	64 (100)	54 (100)	
Other race/ethnicity	1 (1.5)	0	0	
CSF diversion present, *n* (%)	32 (47.1)	4 (6.2)	3 (5.6)	<0.001

**Table 2:** Baseline CT characteristics of the study groups.

Characteristic	Hydrocephalus *n* (%)	Stroke *n* (%)	Trauma *n* (%)	*p*-Value
Periventricular edema	44 (64.7)	26 (40.6)	26 (48.1)	0.015
Intracerebral hemorrhage	1 (1.5)	6 (9.4)	23 (42.6)	<0.001
Subdural hematoma	1 (1.5)	3 (4.7)	35 (64.8)	<0.001
Epidural hematoma	0 (0)	1 (1.6)	15 (27.8)	<0.001
Subarachnoid hemorrhage	1 (1.5)	0 (0)	18 (33.3)	<0.001
Intraventricular hemorrhage	0 (0)	4 (6.3)	10 (18.5)	<0.001
Cerebral atrophy present, *n* (%)	27 (39.7)	28 (43.8)	4 (7.4)	<0.001
Atrophy severity—mild, *n* (%)	16 (59.3)	17 (26.6)	3 (5.6)	0.002
Atrophy severity—moderate, *n* (%)	8 (29.6)	9 (14.1)	1 (1.9)	0.03
Atrophy severity—severe, *n* (%)	3 (11.1)	2 (3.1)	0 (0.0)	0.25
Small vessel disease present, *n* (%)	22 (32.4)	31 (48.4)	32 (59.3)	0.009
Midline shift present, *n* (%)	4 (5.9)	7 (10.9)	24 (44.4)	<0.001

**Table 3:** Linear ventricular parameters across study groups.

Variable	Hydrocephalus (*n* = 68)	Stroke (*n* = 64)	Trauma (*n* = 54)	*p*-Value
Evans index, mean ± SD	0.43 ± 0.07	0.34 ± 0.06	0.33 ± 0.05	<0.001
Third ventricle width (mm), mean ± SD	22.6 ± 5.8	9.1 ± 3.0	9.8 ± 2.7	<0.001
Temporal horn dilation, *n* (%)	30 (44.6)	36 (56.3)	25 (48.1)	0.43
Periventricular edema, *n* (%)	44 (64.7)	26 (40.6)	26 (48.1)	0.015

**Table 4:** Performance of the primary classification model (hydrocephalus vs. non-hydrocephalus).

Metric	Value
ROC AUC	0.71
Brier score	0.20
Accuracy (threshold 0.50)	0.68
Sensitivity (threshold 0.50)	0.34
Specificity (threshold 0.50)	0.87
Youden threshold	0.41
Sensitivity (Youden)	0.69
Specificity (Youden)	0.76

**Table 5:** Multivariable logistic regression predictors of hydrocephalus.

Predictor	Odds Ratio (OR)	95% CI	Interpretation
Evans index (per 0.1 increase)	1.40	1.01–1.99	Strongest ventricular predictor
Third ventricle width (per mm)	1.08	1.01–1.16	Independent morphometric signal
Temporal horn dilation (yes)	0.78	0.42–1.30	Non-specific overlap feature
Periventricular edema (yes)	1.85	1.06–3.55	Supportive CSF flow marker
Age (per year)	0.98	0.96–0.99	Contextual demographic modifier
Cerebral atrophy (present)	0.96	0.47–2.00	Non-independent contextual factor
Midline shift ≥ 1 mm	0.15	0.08–0.28	Distortion marker (non-hydrocephalus)
Mass lesion present	0.66	0.31–1.34	Secondary deformation indicator
Any hemorrhage present	0.08	0.05–0.17	Strong mimic feature

## Data Availability

Data is available on request from the corresponding author.
